# Complement Inhibition in ANCA-Associated Vasculitis

**DOI:** 10.3389/fimmu.2022.888816

**Published:** 2022-07-08

**Authors:** Vladimir Tesar, Zdenka Hruskova

**Affiliations:** Department of Nephrology, 1st Faculty of Medicine, Charles University and General University Hospital in Prague, Prague, Czechia

**Keywords:** ANCA, ANCA - associated vasculitis, complement, avacopan, C5a

## Abstract

Efficacy of immunosuppressive treatment of Anti-neutrophil cytoplasmic antibody (ANCA)-associated vasculitis (AAV) is complicated by its toxicity. With the replacement of cyclophosphamide with rituximab, serious adverse events seem to be associated especially with high-dose corticosteroids. Activation of alternative complement pathway plays an important role in the pathogenesis of AAV. Avacopan (C5a receptor inhibitor) was demonstrated to have at least similar efficacy and better safety (in terms of corticosteroid-related adverse events) compared with high-dose corticosteroids in the induction treatment of AAV. Other modes of the inhibition of alternative complement pathway are currently tested in AAV or could be considered on the basis of the experience in other glomerular diseases.

## Introduction - Efficacy and Toxicity of the Standard Treatment of AAV

Introduction of cyclophosphamide dramatically improved the outcome of ANCA-associated vasculitis (AAV), and patients with AAV were shown to die much less of active vasculitis compared with infection and cardiovascular disease (1). High rate of secondary malignancies in cyclophosphamide-treated patients (2) may be successfully reversed by the replacement of cyclophosphamide with rituximab (3), but the rate of serious adverse events, namely, infections, is similar in patients with active AAV treated with either cyclophosphamide or rituximab (4) possibly due to high-dose corticosteroids used in both induction regimens. Cumulative dose of corticosteroids used in the induction treatment of renal AAV can be safely reduced without any impact on the efficacy of the treatment and with the reduced rate of infections (5, 6). However, it has been demonstrated that the chronic toxicity of corticosteroid treatment starts with much lower doses only slightly above 5 mg of prednisone/day (7). The aim of the treatment should thus be not only to reduce but also to best completely avoid corticosteroid treatment. Recent data suggest that it could be achieved with complement inhibition.

## Role of Complement in the Pathogenesis of AAV

Until quite recently, pathogenesis of AAV was explained by the interplay of genetic predisposition and environmental factors, resulting in loss of tolerance and autoantibody production with subsequent organ injury mediated by neutrophils and monocytes and modified by T cells and macrophages without any clear role to be played by complement (8).

Although it has been always stressed that vasculitis and glomerulonephritis in AAV is pauciimmune, glomerular deposits of C3 were found in glomeruli in more than half of patients with Wegener´s granulomatosis as early as in 1980s (9). Patients with AAV and immune deposits were shown to have higher proteinuria and higher percentage of crescents (10). This observation was confirmed quite recently (11). Patients with glomerular C3d positivity had higher percentage of crescents and lower percentage of normal glomeruli. Glomerular deposition of C3d and properdin was more pronounced in patients with higher proteinuria and higher proportion of crescents (11).

Serum levels of C3 below median (still in the normal range) were also associated in patients with AAV with renal function at diagnosis and worse patient and renal survival (12). Serum C3 levels negatively correlated with the percentage of glomeruli with cellular crescents (12). Association of lower serum C3 levels with patient and renal survival and relapse-free survival was also confirmed in other studies (13, 14).

Importantly, this association was not shown for serum C4 levels, suggesting that C3 is not activated through classical pathway (12). On the other hand, patients with active AAV had increased serum levels of not only C3a, C5a, and C5b-9 but also Bb, suggesting the role of alternative complement pathway (15). Serum Bb levels correlated with erythrocyte sedimentation rate, BVAS (Birmingham Vasculitis Activity Score), and proportion of glomeruli with (cellular) crescents. Importantly, all these markers decreased significantly after treatment and in patients with AAV in remission were no longer significantly different from healthy controls (15).

In keeping with these observations in patients with active AAV, the proportion of glomeruli with crescents correlated positively and the proportion of normal glomeruli correlated negatively with the renal expression of Bb (16). Low levels of serum C3c were shown to correlate in AAV with the severity of kidney impairment at the time of renal biopsy and with interstitial vasculitis (17).

Circulating concentrations of C3a, C5a, factor B, and C5b-9 were higher in patients with AAV compared with healthy controls, and in the remission serum levels of C3a, C5a, and factor B decreased, whereas that of C5b-9 did not (18). Activation of alternative complement pathway was also confirmed by the meta-analysis of five similar studies (18).

Complement activation (including increased serum levels of C5 and C5a) seems to precede clinical symptoms of AAV by several years. Increase of C5 seems to be associated with future development of anti-MPO AAV and renal involvement (19). On the other hand, increased serum levels of C5a were described in patients with both anti-MPO and anti-PR3 antibodies and were significantly lower in patients in long-term remission (20).

## Complement Inhibition in AAV—*In Vitro* and *In Vivo* Experimental Studies

Supernatants from ANCA-activated neutrophils activate complement and produce C5a with subsequent respiratory burst, which can be blocked by the inhibition of C5a receptor, but not C3a receptor. Priming of neutrophils with C5a, but not C3a, dose-dependently stimulates ANCA-induced respiratory burst (21). Furthermore, neutrophils from patients with AAV primed by C5a and stimulated with ANCA have increased potential to activate alternative complement pathway compared with primed neutrophils from healthy subjects (22).

Rapid depletion of C3 induced by cobra venom factor resulted in the amelioration of the course of experimental anti-MPO glomerulonephritis. Mice with experimental anti-MPO glomerulonephritis treated with cobra venom factor had no infiltration of glomeruli with neutrophils and macrophages (23). No necroses and no crescents were observed in mice with experimental anti-MPO glomerulonephritis knockout for C5 and factor B, but not for C4 (23).

Pretreatment of rats with experimental anti-MPO glomerulonephritis with anti-C5 monoclonal antibody significantly reduced early glomerular neutrophil influx and prevented the necroses and crescent formation (24). Administration of anti-C5 antibodies ameliorated the course of the disease (development of necrotizing/crescentic glomerulonephritis) even in mice with already established anti-MPO glomerulonephritis.

There are two receptors of C5a. CD88 is the main C5a receptor and mediator of C5a action on neutrophils. The role of second C5a receptor (C5L2) remains unclear with somewhat conflicting data on the effect of its blockade in AAV (25, 26). C5a receptor was shown to be implicated in crescent formation (21). Mice knockout for CD88 C5a receptor with experimental anti-MPO glomerulonephritis did not develop any necroses and crescents.

CCX168 (avacopan) is an oral inhibitor of C5a receptor (27). CCX168 dose-dependently blocked the migration of neutrophils into the tissues. In experimental anti-MPO glomerulonephritis, CCX168 decreased proteinuria, hematuria, and leukocyturia and dose-dependently decreased the percentage of glomerular crescents (25).

## Complement Inhibition in AAV—Clinical Studies

Non-selective inhibition of complement with anti-C5 antibody (eculizumab) was shown in a case report to improve renal function in the patient with AAV (28) and was effective in a patient with severe AAV in combination with rituximab in corticosteroid-free regimen (29).

**Figure 1:**
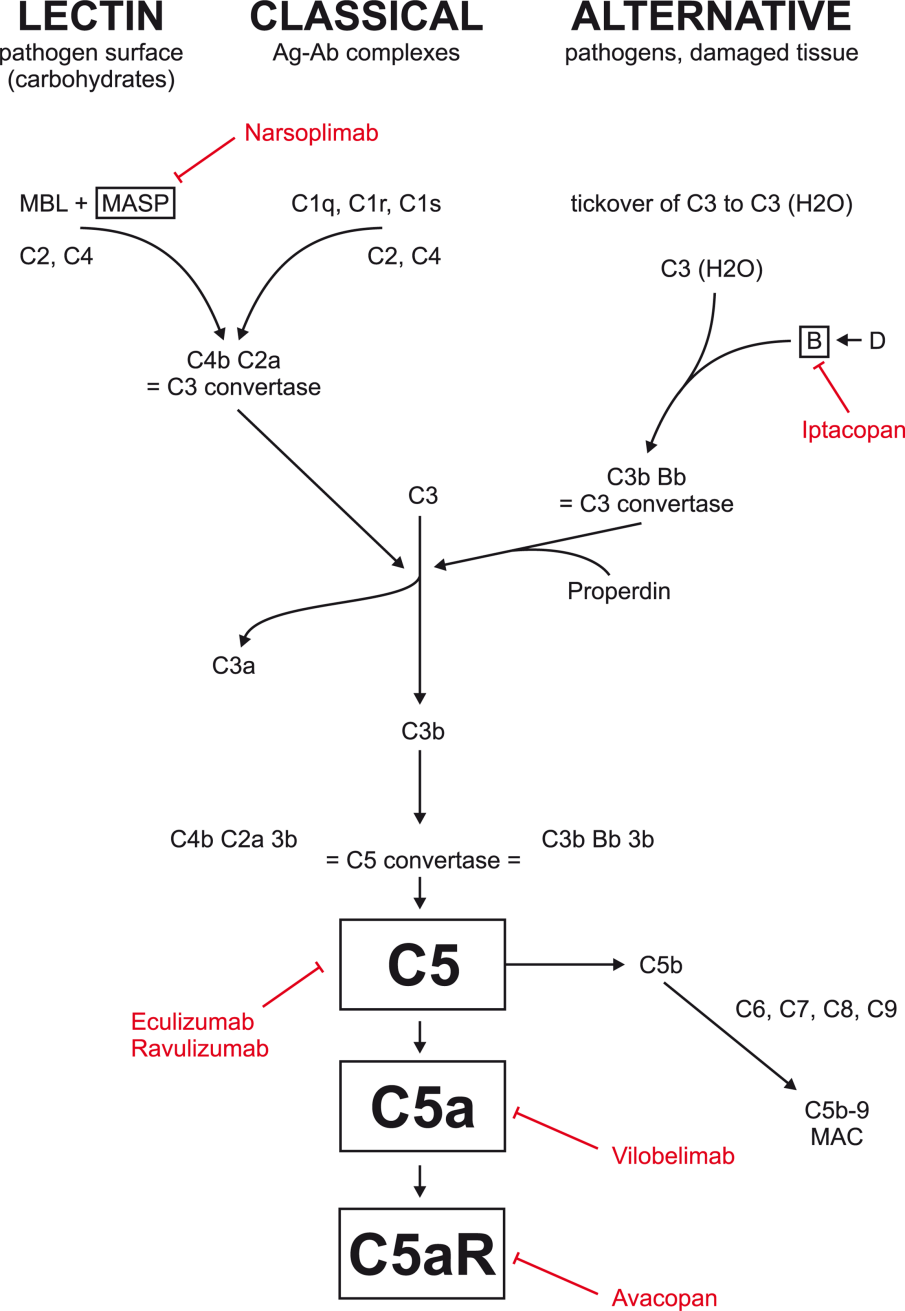
Complement inhibition currently tested in glomerular disease. Eculizumab and ravulizumab are antibodies against factor C5 used primarily in atypical hemolytic uremic syndrome. Avacopan is an oral inhibitor of C5a receptor. Vilobelimab is an antibody against factor C5a. Narsoplimab is antibody against MASP and is currently tested in IgA nephropathy. Iptacopan is an oral factor B inhibitor currently tested in IgA nephropathy, membranous nephropathy, and C3 glomerulopathy.

Inhibition of C5 blocks the formation not only of C5a but also of membrane attack complex (MAC; **Figure 1**) and may be associated with higher risk of bacterial infections requiring, e.g., vaccination against Neisseria meningitidis. More selective inhibition of C5a only, or factor B, could be safer as the activation of MAC through classical and lectin pathway may proceed unhindered.

On the basis of the experimental evidence that inhibition of C5a receptor may ameliorate the course of experimental AAV (30), phase 2 trial (CLEAR) with avacopan in patients with AAV was initiated (31).

CLEAR trial recruited 67 adult patients with newly diagnosed or relapsing AAV [both granulomatosis with polyangiitis (GPA) and microscopic polyangiitis (MPA)] with estimated glomerular filtration rate (eGFR) at least 20 ml/min/1.73 m^2^ that required treatment with cyclophosphamide of rituximab. Patients with severe disease (namely, with rapidly progressive glomerulonephritis or alveolar haemorrhage with severe hypoxia), patients recently treated with cyclophosphamide or rituximab, or patients pretreated with higher dose of corticosteroids (cumulative dose of intravenous methylprednisolone greater than 3 grams the last 3 months, or with oral corticosteroids at a dose higher than 10 mg of prednisone or its equivalent for more than 6 weeks) were excluded.

Patients were randomized into three limbs (placebo with 60 mg of prednisone, 30 mg of avacopan twice daily plus 20 mg of prednisone, and avacopan without prednisone) and followed for 12 weeks. Almost all patients had renal involvement, but they had well preserved renal function with mean eGFR around 50 ml/min/1.73 m^2^.

In both avacopan limbs, there was more rapid decrease of the activity of the disease (measured as BVAS) and more substantial decrease of albuminuria. Patients treated with avacopan without corticosteroids had better quality of life, significant improvement in vitality (fatigue), and lower rate of adverse events, especially adverse events related to corticosteroid treatment (psychiatric disorders, new onset/worsening diabetes, weight gain, fractures, and cataracts), suggesting that avacopan could be a useful steroid sparing drug for patients with AAV.

In another phase 2 trial (CLASSIC) aimed, namely, at the evaluation of safety of avacopan (32), 42 patients with newly diagnosed AAV treated with corticosteroids with either cyclophosphamide or rituximab were randomized either to standard care only or to avacopan (10 or 30 mg twice daily) as add-on treatment. The rate of serious adverse events did not differ between patients treated with standard treatment only and patients receiving avacopan. Higher dose of avacopan was numerically better than placebo and lower dose of avacopan in terms of inducing early remission, improved estimated glomerular filtration rate, renal response, and quality of life.

Subsequent phase 3 trial (ADVOCATE; 33) recruited 331 patients with new or relapsing AAV (both GPA and MPA and both anti-PR3 and anti-MPO positive) with at least one major, or at least three non-major, or at least two renal (proteinuria and hematuria) BVAS items and eGFR ≥ 15 ml/min/1.73 m^2^ (mean eGFR was around 45 ml/min/1.73m^2^) indicated to the treatment with rituximab or cyclophosphamide. Patients were randomized to either high-dose corticosteroids (as in CLEAR study) gradually tapered and completely discontinued by week 21 or avacopan (30 mg twice daily) for 52 weeks.

Primary end points were remission defined as BVAS score of zero at week 26 and sustained remission at week 52. Secondary end points included adverse events, glucocorticoid toxicity, rapidity of response, and health-related quality of life.

BVAS remission at week 26 was achieved in 72.3% of the avacopan-treated subjects vs. 70.1% of subjects in the glucocorticoid standard of care (SOC) control group (p < 0.001 for non-inferiority). Sustained remission at week 52 was observed in 65.7% of the avacopan-treated subjects vs. 54.9% in the glucocorticoid SOC control group (p = 0.0066 for superiority of avacopan). Relapse rate was lower in avacopan limb (10.1%) compared with patients treated with corticosteroids (21%) although it may have been partly related to earlier withdrawal of corticosteroids compared with avacopan.

Additional benefits in avacopan-treated patients included significant improvement in kidney function in patients with renal disease [increase of eGFR was higher in the avacopan-treated patients both at 26 (5.8 vs. 2.8 ml/min/1.73 m^2^, p = 0.0413) and 52 weeks (7.3 vs. 4.0 ml/min/1.73 m^2^, p = 0.0259)]. It remains, however, uncertain, to what extent the difference in eGFR at 1 year in the avacopan limb may also be related to the prolongation of avacopan treatment until 12 months compared with corticosteroid withdrawal already by 6 months.

The number of fatal infections, life-threatening infections, and serious infections was fairly similar in the avacopan and control groups. Importantly, the occurrence of adverse events possibly related to glucocorticoids was 66.3% in the avacopan group and 80.5% in the prednisone group. Avacopan-treated patients showed significant reduction in glucocorticoid-related toxicity (Glucocorticoid Toxicity Index, p < 0.0002 for superiority of avacopan) and statistically significant improvement in health-related quality of life. On the basis of these data in October 2021, avacopan was approved in the USA as an adjunctive treatment in adults for severe active ANCA-associated vasculitis (specifically MPA and GPA) in combination with standard therapy including glucocorticoids (as avacopan did not completely eliminate glucocorticoid use although the total dose of non-study glucocorticoids was higher in prednisone limb compared with avacopan limb). Avacopan was also approved in Japan (in September 2021), has received a positive opinion in the European Union (EU), and is under evaluation in Switzerland and Canada (34). Recently, positive safety data from phase 2 trial with anti-C5a antibody vilobelimab in patients with AAV (IXCHANGE - NCT03895801) were reported (tml, https://www.inflarx.de/Home/Investors/Press-Releases/11-2021-InflaRx-Announces-Positive-Data-from-Phase-II-IXCHANGE-Study-with-Vilobelimab-in-ANCA-associated-Vasculitis--AAV-.html, https://www.inflarx.de/Home/Investors/Press-Releases/11-2021-InflaRx-Announces-Positive-Data-from-Phase-II-IXCHANGE-Study-with-Vilobelimab-in-ANCA-associated-Vasculitis–AAV-.html ). In this study in 57 European patients with AAV, vilobelimab (800 mg each 2 weeks for 16 weeks) was shown to be comparable to standard care with high-dose corticosteroids (as add-on treatment to cyclophosphamide or rituximab) in clinical response and Vasculitis Damage Index with lower composite score of glucocorticoid toxicity index and lower rate of treatment emergent adverse events in vilobelimab limb. Earlier last year, positive data from another phase 2 trial (US IXPLORE, NCT 03712345) demonstrated safety of vilobelimab if given as add-on treatment to standard care (https://www.globenewswire.com/en/news-release/2021/05/11/2227670/0/en/InflaRx-Announces-Positive-Topline-Results-for-Vilobelimab-from-the-U-S-Phase-II-ANCA-Associated-Vasculitis-IXPLORE-Study.html). These promising data are to be confirmed in phase 3 trial. As activated factor B seems to play an important role in the pathogenesis of ANCA-associated glomerulonephritis (23), inhibition of complement factor D that is activating factor B could also be a plausible therapeutic option not only in IgA nephropathy but also in AAV (35, **Figure 1**).

## Conclusions

Replacement of corticosteroids with C5a receptor inhibitor avacopan is already a feasible therapeutic option that should decrease the corticosteroid-related toxicity and could even improve long-term outcome of patients with AAV (lower risk of relapses, better renal function).

Despite encouraging results, many questions remain unanswered, e.g., what is the efficacy and safety of avacopan in patients with advanced renal failure and more severe disease, what is the effect of avacopan on extrarenal (including granulomatous) manifestations of the disease, which patients with AAV may benefit from avacopan treatment most, or if there is any difference in response to avacopan between anti-PR3 and anti-MPO patients. We also do not know what would be the long-term outcome of avacopan-treated patients (in terms of mortality, damage, risk of end-stage kidney disease, etc.). It is also unclear whether avacopan could be used (possibly in lower dose) as a potential maintenance treatment. In our opinion, avacopan may help to avoid unnecessary corticosteroid-related toxicity and should be used, namely, on one hand, in frail elderly patients with comorbidities, or in patients with obesity and diabetes, and, on the other hand, in younger patients, especially with impending repeated exposure to corticosteroids because of relapses. A very important question that will have significant impact on the routine availability of avacopan for patients with AAV will also be its cost effectiveness (36). Real-world experience will help to define the place of avacopan in the treatment armamentarium.

## Author Contributions

Both authors contributed to the selection of papers and design and writing of the paper. Both authors contributed to the article and approved the submitted version.

## Funding

This paper was supported by the research initiative of the Ministry of Health of Czech Republic RVO-VFN 64165.

## Conflict of Interest

VT was principal investigator in both CLEAR and ADVOCATE trials with avacopan. The remaining author declares that the research was conducted in the absence of any commercial or financial relationships that could be construed as a potential conflict of interest.

## Publisher’s Note

All claims expressed in this article are solely those of the authors and do not necessarily represent those of their affiliated organizations, or those of the publisher, the editors and the reviewers. Any product that may be evaluated in this article, or claim that may be made by its manufacturer, is not guaranteed or endorsed by the publisher.
